# Supplementary material for: A gendered content analysis of the World Health Organization’s COVID-19 guidance and policies
Source: PLOS Glob Public Health. 2022 Jun 30;2(6):e0000640. doi: 10.1371/journal.pgph.0000640 (PMC10021261; doi:10.1371/journal.pgph.0000640)
Supplement: S1 Appendix — (DOCX) [file pgph.0000640.s001.docx]

| **Date on document** | **Title** | **Source (Link)** |
| --- | --- | --- |
| 1-Jan-20 | Risk communication and community engagement readiness and initial response for novel coronaviruses (nCoV), v1 draft | <https://apps.who.int/iris/bitstream/handle/10665/330377/WHO-2019-nCoV-RCCE-v2020.1-eng.pdf?sequence=1&isAllowed=y> |
| 9-Jan-20 | Disease Commodity Packages, v1 | <https://apps.who.int/iris/handle/10665/332316> |
| 10-Jan-20 | Laboratory testing of human suspected cases of novel coronavirus (nCoV) infection, v1 | <https://www.who.int/publications/i/item/10665-330374> |
| 10-Jan-20 | National capacities review tool for a novel coronavirus | <https://www.who.int/publications/i/item/national-capacities-review-tool-for-a-novelcoronavirus> |
| 10-Jan-20 | Risk communication and community engagement readiness and initial response for novel coronaviruses (nCoV), v1 | <https://www.who.int/publications/i/item/risk-communication-and-community-engagement-readiness-and-initial-response-for-novel-coronaviruses-(ncov)> |
| 11-Jan-20 | Surveillance case definitions for human infection with novel coronavirus (nCoV), v1 | <https://apps.who.int/iris/handle/10665/330376> |
| 12-Jan-20 | Clinical management of severe acute respiratory infection when novel coronavirus (nCoV) infection is suspected, v1 | https://apps.who.int/iris/handle/10665/332299?search-result=true&query=clinical+management+of+severe+acute+respiratory+infection+when+novel+coronavirus&scope=&filtertype_0=dateIssued&filter_relational_operator_0=contains&filter_0=2020&rpp=10&sort_by=score&order=desc |
| 14-Jan-20 | Laboratory testing for 2019 novel coronavirus (2019-nCoV) in suspected human cases, v2 | <https://apps.who.int/iris/handle/10665/332300> |
| 15-Jan-20 | Surveillance case definitions for human infection with novel coronavirus (nCoV), v2 | <https://apps.who.int/iris/handle/10665/332412> |
| 17-Jan-20 | Laboratory testing of 2019 novel coronavirus (‎‎‎‎‎‎‎‎‎‎‎‎2019-nCoV)‎‎‎‎‎‎‎‎‎‎‎‎ in suspected human cases, v3 | https://apps.who.int/iris/handle/10665/330676?search-result=true&query=laboratory+testing+of+2019+novel+coronavirus&scope=&rpp=10&sort_by=score&order=desc |
| 20-Jan-20 | Home care for patients with suspected novel coronavirus (‎‎‎‎‎‎‎‎‎‎‎nCoV)‎‎‎‎‎‎‎‎‎‎‎ infection presenting with mild symptoms and management of their contacts, v1 | <https://www.who.int/publications/i/item/10665-330671> |
| 20-Jan-20 | Outline of designs for experimental therapeutics, v2 | <https://www.who.int/publications/i/item/outline-of-designs-for-experimental-vaccines-and-therapeutics-20-january-2020> |
| 21-Jan-20 | Data dictionary for case reporting form, v1 | <https://apps.who.int/iris/handle/10665/332413> |
| 21-Jan-20 | Global surveillance for human infection with novel coronavirus (‎2019-nCoV)‎, v1 | <https://apps.who.int/iris/handle/10665/336097> |
| 21-Jan-20 | Interim case reporting form for 2019 Novel Coronavirus (‎2019-nCoV)‎ of confirmed and probable cases, v1 | <https://apps.who.int/iris/handle/10665/332411> |
| 21-Jan-20 | Outline of designs for experimental therapeutics, v5 | <https://apps.who.int/iris/handle/10665/330695> |
| 22-Jan-20 | Surveillance line list, v1 | <https://apps.who.int/iris/handle/10665/332414> |
| 24-Jan-20 | Informal consultation on prioritization of candidate therapeutic agents for use in novel coronavirus 2019 infection | <https://www.who.int/publications/i/item/informal-consultation-on-prioritization-of-candidate-therapeutic-agents-for-use-in-novel-coronavirus-2019-infection> |
| 24-Jan-20 | Molecular assays to diagnose COVID-19: Summary table of available protocols | <https://www.who.int/publications/m/item/molecular-assays-to-diagnose-covid-19-summary-table-of-available-protocols> |
| 24-Jan-20 | Prospects for evaluating cross-reactivity of nCoV with SARS-CoV, v3 | <https://www.who.int/publications/i/item/who-r-d-blueprint-novel-coronavirus-prospects-for-evaluating-cross-reactivity-of-ncov-with-sars-cov> |
| 25-Jan-20 | Clinical management of severe acute respiratory infection when novel coronavirus (nCoV) infection is suspected, v2 | https://apps.who.int/iris/bitstream/handle/10665/330854/WHO-nCoV-Clinical-2020.2-eng.pdf?sequence=1&isAllowed=y |
| 25-Jan-20 | Infection prevention and control during health care when novel coronavirus (nCoV) infection is suspected, v1 | <https://apps.who.int/iris/handle/10665/330674> |
| 26-Jan-20 | Risk communication and community engagement (RCCE) readiness and response to the 2019 novel coronavirus (2019-nCoV) | <https://apps.who.int/iris/handle/10665/330678> |
| 27-Jan-20 | Disease Commodity Packages, v2 | <https://apps.who.int/iris/handle/10665/332317> |
| 27-Jan-20 | Outline of trial designs for experimental therapeutics, v4 | <https://www.who.int/publications/i/item/outline-of-trial-designs-for-experimental-therapeutics> |
| 27-Jan-20 | WHO recommendations to reduce risk of transmission of emerging pathogens from animals to humans in live animal markets or animal product markets, v1 | <https://apps.who.int/iris/handle/10665/332216> |
| 28-Jan-20 | Clinical management of severe acute respiratory infection when novel coronavirus (nCoV) infection is suspected, v3 | https://apps.who.int/iris/handle/10665/330893 |
| 29-Jan-20 | Advice on the use of masks in the community, during home care, and in health care settings in the context of the novel coronavirus (2019-nCOV) outbreak, v1 | <https://apps.who.int/iris/handle/10665/330987> |
| 30-Jan-20 | Vaccine prioritization for clinical trials | <https://www.who.int/publications/i/item/who-r-d-blueprint-novel-coronavirus-ncov-vaccine-prioritization-for-clinical-trials> |
| 31-Jan-20 | Global surveillance for human infection with novel coronavirus (‎‎‎‎2019-nCoV)‎‎‎‎, v3 | <https://apps.who.int/iris/handle/10665/330857> |
| 3-Feb-20 | Strategic Preparedness and Response Plan (draft as of 3-Feb-20) | https://www.who.int/docs/default-source/coronaviruse/srp-04022020.pdf |
| 4-Feb-20 | Home care for patients with suspected novel coronavirus (‎‎‎‎‎‎‎‎‎‎‎nCoV)‎‎‎‎‎‎‎‎‎‎‎ infection presenting with mild symptoms and management of their contacts, v2 | <https://apps.who.int/iris/handle/10665/331133> |
| 4-Feb-20 | Strategic Preparedness and Response Plan (SPRP) | <https://www.who.int/publications/i/item/strategic-preparedness-and-response-plan-for-the-new-coronavirus> |
| 7-Feb-20 | Disease Commodity Packages, v3 | <https://apps.who.int/iris/handle/10665/332406> |
| 10-Feb-20 | The first few X cases and contacts (‎FFX)‎ investigation protocol for coronavirus disease 2019 (‎COVID-19) (version 2) | <https://apps.who.int/iris/handle/10665/331235> |
| 12-Feb-20 | Laboratory biosafety guidance related to coronavirus disease (COVID-19), v1 | https://apps.who.int/iris/handle/10665/331138 |
| 12-Feb-20 | Operational planning guidelines to support country preparedness and response, draft as of 12 February 2020 | <https://www.who.int/publications/i/item/draft-operational-planning-guidance-for-un-country-teams> |
| 14-Feb-20 | Key planning recommendations for mass gatherings in the context of the current COVID-19 outbreak, v1 | <https://apps.who.int/iris/handle/10665/331004> |
| 16-Feb-20 | Management of ill travellers at points of entry (international airports, seaports and ground crossings) in the context of COVID -19 outbreak, v1 | <https://apps.who.int/iris/handle/10665/331003> |
| 18-Feb-20 | COVID-19 Therapeutic Trial Synopsis | <https://www.who.int/publications/i/item/covid-19-therapeutic-trial-synopsis> |
| 18-Feb-20 | Surface sampling of coronavirus disease (‎COVID-19)‎: a practical “how to” protocol for health care and public health professionals (version 1.1) | <https://www.who.int/publications/i/item/surface-sampling-of-coronavirus-disease-(-covid-19)-a-practical-how-to-protocol-for-health-care-and-public-health-professionals> |
| 23-Feb-20 | The first few X cases and contacts (‎FFX)‎ investigation protocol for coronavirus disease 2019 (‎COVID-19) (version 2.2) | <https://www.who.int/publications/i/item/the-first-few-x-cases-and-contacts-(-ffx)-investigation-protocol-for-coronavirus-disease-2019-(-covid-19)-version-2.2> |
| 24-Feb-20 | COVID-19 Early Epidemiologic and Clinical investigations for public health response | <https://www.who.int/publications/m/item/covid-19-early-epidemiologic-and-clinical-investigations-for-public-health-response> |
| 24-Feb-20 | Operational considerations for managing COVID-19 cases or outbreaks on board ships, v1 | https://apps.who.int/iris/handle/10665/331164 |
| 24-Feb-20 | Social stigma associated with COVID-19 | <https://www.who.int/publications/m/item/a-guide-to-preventing-and-addressing-social-stigma-associated-with-covid-19> |
| 27-Feb-20 | Data dictionary for case reporting form, v2 | <https://www.who.int/publications/m/item/data-dictionary-for-case-based-reporting-form> |
| 27-Feb-20 | Global surveillance for COVID-19 disease caused by human infection with novel coronavirus (‎‎‎‎‎‎‎COVID-19)‎‎‎‎‎‎‎, v4 | <https://apps.who.int/iris/handle/10665/331231> |
| 27-Feb-20 | Rational use of personal protective equipment for coronavirus disease (‎‎‎‎COVID-19)‎‎‎‎, v1 | <https://apps.who.int/iris/handle/10665/331215> |
| 27-Feb-20 | Surveillance line list, v2 | <https://apps.who.int/iris/handle/10665/336100> |
| 28-Feb-20 | CRF for Confirmed Novel Coronavirus COVID-19 (report to WHO within 48 hours of case identification), v2 | <https://www.who.int/publications/i/item/revised-case-report-form-for-confirmed-novel-coronavirus-covid-19-(-report-to-who-within-48-hours-of-case-identification)> |
| 28-Feb-20 | Report of the WHO-China Joint Mission on Coronavirus Disease 2019 (COVID-19) | <https://www.who.int/publications/i/item/report-of-the-who-china-joint-mission-on-coronavirus-disease-2019-(covid-19)> |
| 29-Feb-20 | Considerations for quarantine of individuals in the context of containment for coronavirus disease (COVID-19), v1 | https://apps.who.int/iris/handle/10665/331299 |
| 2-Mar-20 | Guidance for laboratories shipping specimens to WHO reference laboratories that provide confirmatory testing for COVID-19 virus, v2 | https://apps.who.int/iris/handle/10665/331337?search-result=true&query=&scope=%2F&filtertype_0=author&filtertype_1=subject&filtertype_2=dateIssued&filter_relational_operator_1=contains&filtertype_3=title&filter_relational_operator_0=equals&filter_2=2020&filter_relational_operator_3=contains&filter_1=COVID-19&filter_relational_operator_2=contains&filter_0=World+Health+Organization&filter_3=guidance+for+laboratories+shipping+specimens&rpp=10&sort_by=dc.date.accessioned_dt&order=desc |
| 2-Mar-20 | Laboratory testing for 2019 novel coronavirus (2019-nCoV) in suspected human cases, v4 | https://apps.who.int/iris/handle/10665/331329 |
| 3-Mar-20 | Water, sanitation, hygiene, and waste management for the COVID-19 virus, v1 | <https://apps.who.int/iris/handle/10665/331305> |
| 4-Mar-20 | Health worker exposure assessment tool - data dictionary | <https://www.who.int/publications/m/item/data-dictionary-for-health-worker-exposure-assessment-tool> |
| 4-Mar-20 | Health worker exposure assessment tool - data template | <https://www.who.int/publications/m/item/data-template-for-health-worker-exposure-assessment-tool> |
| 4-Mar-20 | Health worker exposure risk assessment and management in the context of COVID-19 virus | https://apps.who.int/iris/handle/10665/331340 |
| 4-Mar-20 | Shipment booking form for COVID-19 clinical samples version 2 | <https://www.who.int/publications/m/item/shipment-booking-form-for-covid-19-clinical-samples-version-2> |
| 6-Mar-20 | Disease Commodity Packages, v4 |  |
| 7-Mar-20 | Critical preparedness, readiness and response actions for COVID-19, v1 | <https://apps.who.int/iris/handle/10665/331422> |
| 7-Mar-20 | Responding to community spread of COVID-19 | <https://www.who.int/publications/i/item/responding-to-community-spread-of-covid-19> |
| 10-Mar-20 | Key Messages and Actions for COVID-19 Prevention and Control in Schools | <https://www.who.int/publications/m/item/key-messages-and-actions-for-covid-19-prevention-and-control-in-schools> |
| 12-Mar-20 | A Coordinated Global Research Roadmap | <https://www.who.int/publications/m/item/a-coordinated-global-research-roadmap> |
| 13-Mar-20 | Clinical management of severe acute respiratory infection (SARI) when COVID-19 is suspected, v4 | https://apps.who.int/iris/handle/10665/331446?search-result=true&query=10665%2F331446&scope=&rpp=10&sort_by=score&order=desc |
| 13-Mar-20 | Considerations in the investigation of cases and clusters of COVID-19, v1 | <https://apps.who.int/iris/handle/10665/331447> |
| 13-Mar-20 | Informal consultation on the potential role of chloroquine in the clinical management of COVID 19 infection | <https://www.who.int/publications/i/item/informal-consultation-on-the-potential-role-of-chloroquine-in-the-clinical-management-of-covid-19-infection> |
| 16-Mar-20 | RCCE Action Plan Guidance COVID-19 Preparedness and Response | <https://www.who.int/publications/i/item/risk-communication-and-community-engagement-(rcce)-action-plan-guidance> |
| 17-Mar-20 | Home care for patients with suspected novel coronavirus (‎‎‎‎‎‎‎‎‎‎‎nCoV)‎‎‎‎‎‎‎‎‎‎‎ infection presenting with mild symptoms and management of their contacts, v3 | https://apps.who.int/iris/handle/10665/331473 |
| 17-Mar-20 | Population-based age-stratified seroepidemiological investigation protocol for coronavirus 2019 (COVID-19) infection (version 1.1) | <https://www.who.int/publications/i/item/WHO-2019-nCoV-Seroepidemiology-2020.2> |
| 17-Mar-20 | Scaling up COVID-19 Outbreak Readiness and Response in Camps and Camp Based Settings (jointly developed by IASC / IFRC / IOM / UNHCR / WHO) | <https://www.who.int/publications/m/item/scaling-up-covid-19-outbreak-readiness-and-response-in-camps-and-camp-based-settings-(jointly-developed-by-iasc-ifrc-iom-unhcr-who)> |
| 18-Mar-20 | Mental health and psychosocial considerations during the COVID-19 outbreak | <https://www.who.int/publications/i/item/WHO-2019-nCoV-MentalHealth-2020.1> |
| 18-Mar-20 | Operational considerations for managing COVID-19 cases or outbreak in aviation, v1 | <https://www.who.int/publications/i/item/operational-considerations-for-managing-covid-19-cases-or-outbreak-in-aviation-interim-guidance> |
| 19-Mar-20 | Advice on the use of masks in the community, during home care, and in health care settings in the context of COVID-19, v2 | https://apps.who.int/iris/handle/10665/331493?search-result=true&query=&scope=%2F&filtertype_0=author&filtertype_1=subject&filtertype_2=dateIssued&filter_relational_operator_1=contains&filtertype_3=title&filter_relational_operator_0=equals&filter_2=2020&filter_relational_operator_3=contains&filter_1=COVID-19&filter_relational_operator_2=contains&filter_0=World+Health+Organization&filter_3=advice+on+the+use+of+masks+in+the+community&rpp=10&sort_by=dc.date.accessioned_dt&order=desc |
| 19-Mar-20 | Considerations for quarantine of individuals in the context of containment for coronavirus disease (COVID-19), v2 | https://apps.who.int/iris/handle/10665/331497 |
| 19-Mar-20 | Coronavirus disease (COVID-19) outbreak: rights, roles and responsibilities of health workers, including key considerations for occupational safety and health | https://apps.who.int/iris/bitstream/handle/10665/331510/WHO-2019-nCov-HCWadvice-2020.2-eng.pdf |
| 19-Mar-20 | Critical preparedness, readiness and response actions for COVID-19, v2 | <https://apps.who.int/iris/handle/10665/331494> |
| 19-Mar-20 | Getting your workplace ready for COVID-19: How COVID-19 spreads | <https://www.who.int/publications/m/item/getting-your-workplace-ready-for-covid-19-how-covid-19-spreads> |
| 19-Mar-20 | Infection prevention and control during health care when COVID-19 is suspected, v3 | <https://www.who.int/publications/i/item/10665-331495> |
| 19-Mar-20 | Key planning recommendations for mass gatherings in the context of COVID-19, v2 | <https://apps.who.int/iris/handle/10665/331502> |
| 19-Mar-20 | Laboratory biosafety guidance related to coronavirus disease (COVID-19), v2 |  |
| 19-Mar-20 | Laboratory testing for 2019 novel coronavirus (2019-nCoV) in suspected human cases, v5 | <https://www.who.int/publications/i/item/10665-331501> |
| 19-Mar-20 | Management of ill travellers at points of entry (international airports, seaports and ground crossings) in the context of COVID -19 outbreak, v2 | <https://www.who.int/publications/i/item/10665-331512> |
| 19-Mar-20 | Operational considerations for case management of COVID-19 in health facility and community | <https://www.who.int/publications/i/item/10665-331492> |
| 19-Mar-20 | Rational use of personal protective equipment for coronavirus disease (COVID-19), v2 | <https://www.who.int/publications/i/item/rational-use-of-personal-protective-equipment-(ppe)-for-coronavirus-disease-(covid-19)> |
| 19-Mar-20 | Risk assessment and management of exposure of health care workers in the context of COVID-19 (tool) | <https://www.who.int/publications/i/item/risk-assessment-and-management-of-exposure-of-health-care-workers-in-the-context-of-covid-19-interim-guidance> |
| 19-Mar-20 | Risk communication and community engagement readiness and response to coronavirus disease (COVID-19), v2 | <https://www.who.int/publications/i/item/risk-communication-and-community-engagement-readiness-and-initial-response-for-novel-coronaviruses> |
| 19-Mar-20 | Water, sanitation, hygiene, and waste management for the COVID-19 virus, v2 | <https://apps.who.int/iris/handle/10665/331499> |
| 20-Mar-20 | Global surveillance for COVID-19 caused by human infection with COVID-19 virus, v6 | https://apps.who.int/iris/handle/10665/331506?search-result=true&query=&scope=%2F&filtertype_0=author&filtertype_1=subject&filtertype_2=dateIssued&filter_relational_operator_1=contains&filtertype_3=title&filter_relational_operator_0=equals&filter_2=2020&filter_relational_operator_3=contains&filter_1=COVID-19&filter_relational_operator_2=contains&filter_0=World+Health+Organization&filter_3=global+surveillance+for+covid-19&rpp=10&sort_by=dc.date.accessioned_dt&order=desc |
| 20-Mar-20 | Global surveillance of COVID-19: WHO process for reporting aggregated data, reporting tool v1 | <https://apps.who.int/iris/handle/10665/331563> |
| 20-Mar-20 | How to use the WHO mass gathering religious addendum risk assessment tool in the context of COVID-19 | <https://www.who.int/publications/i/item/how-to-use-the-who-mass-gathering-religious-addendum-risk-assessment-tool-in-the-context-of-covid-19> |
| 20-Mar-20 | How to use WHO risk assessment and mitigation checklist for Mass Gatherings in the context of COVID-19 | <https://www.who.int/publications/i/item/how-to-use-who-risk-assessment-and-mitigation-checklist-for-mass-gatherings-in-the-context-of-covid-19> |
| 20-Mar-20 | Maintaining a safe and adequate blood supply during the pandemic outbreak of coronavirus disease (COVID-19), v1 | https://apps.who.int/iris/handle/10665/331523 |
| 21-Mar-20 | Infection prevention and control guidance for long-term care facilities in the context of COVID-19 | <https://www.who.int/publications/i/item/WHO-2019-nCoV-IPC-long-term-care-2020-1> |
| 21-Mar-20 | Laboratory testing strategy recommendations for COVID-19 | <https://www.who.int/publications/i/item/laboratory-testing-strategy-recommendations-for-covid-19-interim-guidance> |
| 22-Mar-20 | Critical preparedness, readiness and response actions for COVID-19, v3 | https://apps.who.int/iris/handle/10665/331511 |
| 23-Mar-20 | Household transmission investigation protocol for 2019-novel coronavirus (version 2.2) | <https://www.who.int/publications/i/item/household-transmission-investigation-protocol-for-2019-novel-coronavirus-(2019-ncov)-infection> |
| 23-Mar-20 | Protocol for assessment of potential risk factors for 2019-novel coronavirus (COVID-19) infection among health care workers in a health care setting (Version 2.2) | <https://www.who.int/publications/i/item/protocol-for-assessment-of-potential-risk-factors-for-2019-novel-coronavirus-(2019-ncov)-infection-among-health-care-workers-in-a-health-care-setting> |
| 24-Mar-20 | Infection prevention and control for the safe management of a dead body in the context of COVID-19, v1 | <https://apps.who.int/iris/handle/10665/331538> |
| 25-Mar-20 | Operational considerations for managing COVID-19 cases or outbreaks on board ships, v2 | <https://www.who.int/publications/i/item/operational-considerations-for-managing-covid-19-cases-outbreak-on-board-ships> |
| 25-Mar-20 | Operational guidance for maintaining essential health services during an outbreak, v1 | https://apps.who.int/iris/handle/10665/331561 |
| 26-Mar-20 | Disability considerations during the COVID-19 outbreak | <https://www.who.int/publications/i/item/WHO-2019-nCoV-Disability-2020-1> |
| 26-Mar-20 | Guiding principles for immunization activities during the COVID-19 pandemic | <https://apps.who.int/iris/handle/10665/331590> |
| 26-Mar-20 | Operational considerations for COVID-19 surveillance using GISRS | <https://www.who.int/publications/i/item/operational-considerations-for-covid-19-surveillance-using-gisrs-interim-guidance> |
| 26-Mar-20 | Origin of SARS-CoV-2 | <https://www.who.int/publications/i/item/origin-of-sars-cov-2> |
| 26-Mar-20 | WHO recommendations to reduce risk of transmission of emerging pathogens from animals to humans in live animal markets or animal product markets, v2 | <https://www.who.int/publications/i/item/10665332217> |
| 27-Mar-20 | Modes of transmission of virus causing COVID-19: implications for IPC precaution recommendations, v1 | <https://apps.who.int/iris/handle/10665/331601> |
| 28-Mar-20 | Severe Acute Respiratory Infections Treatment Centre | <https://www.who.int/publications/i/item/10665-331603> |
| 29-Mar-20 | Modes of transmission of virus causing COVID-19: implications for IPC precaution recommendations, v2 | <https://apps.who.int/iris/handle/10665/331616> |
| 31-Mar-20 | Go.data User Guide, version 2.0 | <https://apps.who.int/iris/handle/10665/332255> |
| 31-Mar-20 | Guidance for laboratories shipping specimens to WHO reference laboratories that provide confirmatory testing for COVID-19 virus, v3 | <https://www.who.int/publications/i/item/guidance-for-laboratories-shipping-specimens-to-who-reference-laboratories-that-provide-confirmatory-testing-for-covid-19-virus> |
| 31-Mar-20 | Off-label use of medicines for COVID-19 | <https://www.who.int/publications/i/item/off-label-use-of-medicines-for-covid-19-scientific-brief> |
| 31-Mar-20 | Operational considerations for COVID-19 management in the accommodation sector, v1 | <https://apps.who.int/iris/handle/10665/331638> |
| 1-Apr-20 | Recommendations to Member States to improve hand hygiene practices to help prevent the transmission of the COVID-19 virus | <https://www.who.int/publications/i/item/recommendations-to-member-states-to-improve-hand-hygiene-practices-to-help-prevent-the-transmission-of-the-covid-19-virus> |
| 2-Apr-20 | Considerations in the investigation of cases and clusters of COVID-19, v2 | <https://apps.who.int/iris/handle/10665/331668> |
| 4-Apr-20 | Oxygen sources and distribution for COVID-19 treatment centres | <https://www.who.int/publications/i/item/oxygen-sources-and-distribution-for-covid-19-treatment-centres> |
| 5-Apr-20 | SAVE LIVES: Clean Your Hands - in the context of COVID-19 | <https://www.who.int/publications/m/item/save-lives-clean-your-hands-in-the-context-of-covid-19> |
| 6-Apr-20 | Advice on the use of masks in the context of COVID-19, v3 | <https://apps.who.int/iris/handle/10665/331693> |
| 6-Apr-20 | Global surveillance of COVID-19: WHO process for reporting aggregated data, reporting tool v2 | <https://apps.who.int/iris/handle/10665/331696> |
| 6-Apr-20 | Rational use of personal protective equipment for coronavirus disease (‎‎‎‎‎‎‎COVID-19)‎‎‎‎‎‎‎ and considerations during severe shortages, v3 | <https://apps.who.int/iris/handle/10665/331695> |
| 7-Apr-20 | COVID-19 and Food Safety: Guidance for Food Businesses | <https://www.who.int/publications/i/item/covid-19-and-food-safety-guidance-for-food-businesses> |
| 7-Apr-20 | COVID-19 and violence against women: what the health sector/system can do | <https://www.who.int/publications/i/item/covid-19-and-violence-against-women> |
| 7-Apr-20 | Practical considerations and recommendations for religious leaders and faith-based communities in the context of COVID-19 | <https://www.who.int/publications/i/item/practical-considerations-and-recommendations-for-religious-leaders-and-faith-based-communities-in-the-context-of-covid-19> |
| 8-Apr-20 | Advice on the use of point-of-care immunodiagnostic tests for COVID-19 | <https://www.who.int/publications/i/item/advice-on-the-use-of-point-of-care-immunodiagnostic-tests-for-covid-19-scientific-brief> |
| 8-Apr-20 | Assessment tool for laboratories implementing SARS-CoV-2 testing, v1 | <https://apps.who.int/iris/handle/10665/331714> |
| 8-Apr-20 | Assessment tool for laboratories implementing SARS-CoV-2 testing: annex: assessment tool / facility questionaire, v1 | <https://apps.who.int/iris/handle/10665/331715> |
| 8-Apr-20 | Global COVID-19 Clinical Platform: Rapid Case Report Form, v3 | <https://apps.who.int/iris/handle/10665/331768> |
| 8-Apr-20 | Managing the COVID-19 infodemic: call for action | <https://www.who.int/publications/i/item/9789240010314> |
| 9-Apr-20 | List of priority medical devices for COVID-19 case management | <https://www.who.int/publications/m/item/list-of-priority-medical-devices-for-covid-19-case-management> |
| 9-Apr-20 | Priority medical devices in the context of COVID-19, v1 | <https://apps.who.int/iris/handle/10665/336784> |
| 10-Apr-20 | Informal consultation on the potential inclusion of Favipiravir in a clinical trial | <https://www.who.int/publications/i/item/who-r-d-blueprint-covid-19-informal-consultation-on-the-potential-inclusion-of-favipiravir-in-a-clinical-trial> |
| 11-Apr-20 | Clinical care of severe acute respiratory infections – Toolkit | <https://www.who.int/publications/i/item/clinical-care-of-severe-acute-respiratory-infections-tool-kit> |
| 12-Apr-20 | Bacille Calmette-Guérin (BCG) vaccination and COVID-19 | <https://www.who.int/publications/i/item/bacille-calmette-guérin-(bcg)-vaccination-and-covid-19> |
| 14-Apr-20 | Considerations for sports federations/sports event organizers when planning mass gatherings in the context of COVID-19: interim guidance | <https://www.who.int/publications/i/item/considerations-for-sports-federations-sports-event-organizers-when-planning-mass-gatherings-in-the-context-of-covid-19-interim-guidance> |
| 14-Apr-20 | COVID-19 Strategy update (update to the COVID-19 SPRP from 4-Feb-20) | <https://www.who.int/publications/m/item/covid-19-strategy-update> |
| 15-Apr-20 | Safe Ramadan practices in the context of the COVID-19: interim guidance | <https://www.who.int/publications/i/item/safe-ramadan-practices-in-the-context-of-the-covid-19-interim-guidance> |
| 15-Apr-20 | Technical specifications for invasive and non-invasive ventilators for COVID-19 | <https://www.who.int/publications/i/item/technical-specifications-for-invasive-and-non-invasive-ventilaotrs-for-covid-19> |
| 16-Apr-20 | Considerations in adjusting public health and social measures in the context of COVID-19, v1 | <https://apps.who.int/iris/handle/10665/331773> |
| 16-Apr-20 | Immunization in the context of COVID-19 pandemic FAQ | <https://www.who.int/publications/i/item/immunization-in-the-context-of-covid-19-pandemic> |
| 17-Apr-20 | Guidance for the use of the WHO Mass Gatherings Sports: addendum risk assessment tools in the context of COVID-19 | <https://www.who.int/publications/i/item/guidance-for-the-use-of-the-who-mass-gatherings-sports-addendum-risk-assesment-tools-in-the-context-of-covid-19> |
| 17-Apr-20 | Preparedness, prevention and control of coronavirus disease (COVID-19) for refugees and migrants in non-camp settings | <https://www.who.int/publications/i/item/preparedness-prevention-and-control-of-coronavirus-disease-(covid-19)-for-refugees-and-migrants-in-non-camp-settings> |
| 17-Apr-20 | Violence against women and girls data collection during COVID-19 | https://www.who.int/publications/i/item/violence-against-women-and-girls-data-collection-during-covid-19 |
| 19-Apr-20 | The use of non-steroidal anti-inflammatory drugs (NSAIDs) in patients with COVID-19 | <https://www.who.int/publications/i/item/the-use-of-non-steroidal-anti-inflammatory-drugs-(nsaids)-in-patients-with-covid-19> |
| 19-Apr-20 | WHO reference laboratories providing confirmatory testing for COVID-19 | <https://www.who.int/publications/m/item/who-reference-laboratories-providing-confirmatory-testing-for-covid-19> |
| 21-Apr-20 | Addressing Human Rights as Key to the COVID-19 Response | <https://www.who.int/publications/i/item/addressing-human-rights-as-key-to-the-covid-19-response> |
| 22-Apr-20 | COVID-19 and Food Safety: Guidance for competent authorities responsible for national food safety control systems | <https://www.who.int/publications/i/item/covid-19-and-food-safety-guidance-for-competent-authorities-responsible-for-national-food-safety-control-systems> |
| 23-Apr-20 | Water, sanitation, hygiene, and waste management for the COVID-19 virus, v3 | <https://apps.who.int/iris/handle/10665/331846> |
| 23-Apr-20 | Working with Community Advisory Boards for COVID-19 related clinical studies | <https://www.who.int/publications/m/item/working-with-community-advisory-boards-for-covid-19-related-clinical-studies> |
| 24-Apr-20 | “Immunity passports” in the context of COVID-19 - 24 April 2020 | <https://www.who.int/publications/i/item/10665-331866> |
| 25-Apr-20 | FAQ: WHO COVID-19 essential supplies forecasting tool (COVID-19-ESFT) | <https://www.who.int/publications/i/item/10665-333299> |
| 26-Apr-20 | WHO Working Group – Core protocol for therapeutics against COVID-19 (January 2020) | <https://www.who.int/publications/i/item/who-working-group-core-protocol-for-therapeutics-against-covid19> |
| 28-Apr-20 | COVID 19 Experimental treatments | <https://www.who.int/publications/i/item/covid-19-landscape-of-experimental-treatments> |
| 28-Apr-20 | COVID-19 Supply Chain System: Purchasing consortia status as of 28 April 2020 | <https://www.who.int/publications/m/item/covid-19-supply-chain-system-purchasing-consortia-status-as-of-28-april-2020> |
| 28-Apr-20 | SMS message library | <https://www.who.int/publications/i/item/covid-19-message-library> |
| 28-Apr-20 | Strengthening Preparedness for COVID-19 in Cities and Urban Settings | <https://www.who.int/publications/i/item/strengthening-preparedness-for-covid-19-in-cities-and-urban-settings> |
| 29-Apr-20 | COVID-19 Essential Supplies Forecasting Tool, v2 | <https://apps.who.int/iris/handle/10665/333284> |
| 29-Apr-20 | Preparedness for cyclones, tropical storms, tornadoes, floods and earthquakes during the COVID-19 pandemic | <https://www.who.int/publications/i/item/WHO-2019-nCoV-Advisory-Preparedness-2020.1> |
| 30-Apr-20 | Operational considerations for COVID-19 management in the accommodation sector, v2 | <https://apps.who.int/iris/handle/10665/331937> |
| 5-May-20 | Community-based health care, including outreach and campaigns, in the context of the COVID-19 pandemic | <https://www.who.int/publications/i/item/WHO-2019-nCoV-Comm_health_care-2020.1> |
| 6-May-20 | Key criteria for the ethical acceptability of COVID-19 human challenge studies | <https://apps.who.int/iris/handle/10665/331976> |
| 7-May-20 | COVID-19 and the use of angiotensin-converting enzyme inhibitors and receptor blockers | <https://www.who.int/publications/i/item/covid-19-and-the-use-of-angiotensin-converting-enzyme-inhibitors-and-receptor-blockers> |
| 7-May-20 | Public health and social measures for COVID-19 preparedness and response in low capacity and humanitarian settings, Version 1 (Developed by ICRC, IFRC, IOM, NRC, UNICEF, UN-HABITAT, UNHCR, WHO in consultation with IASC members) | <https://www.who.int/publications/m/item/public-health-and-social-measures-for-covid-19-preparedness-and-response-in-low-capacity-and-humanitarian-settings> |
| 10-May-20 | Considerations for public health and social measures in the workplace in the context of COVID-19 (annex to: Considerations in adjusting public health and social measures in the context of COVID-19) | <https://www.who.int/publications/i/item/considerations-for-public-health-and-social-measures-in-the-workplace-in-the-context-of-covid-19> |
| 10-May-20 | Considerations for school-related public health measures in the context of COVID-19 (annex to: Considerations in adjusting public health and social measures in the context of COVID-19), v1 | <https://apps.who.int/iris/handle/10665/332052> |
| 10-May-20 | Contact tracing in the context of COVID-19 | <https://www.who.int/publications/i/item/contact-tracing-in-the-context-of-covid-19> |
| 10-May-20 | Surveillance strategies for COVID-19 human infection | <https://apps.who.int/iris/handle/10665/332051> |
| 11-May-20 | Global COVID-19 Clinical Platform: CRF with Pregnancy Module, v4 | <https://apps.who.int/iris/handle/10665/332072> |
| 12-May-20 | Breastfeeding and COVID-19 for healthcare workers FAQ | <https://www.who.int/publications/m/item/frequently-asked-questions-breastfeeding-and-covid-19> |
| 12-May-20 | Public health criteria to adjust public health and social measures in the context of COVID-19: annex to considerations in adjusting public health and social measures in the context of COVID-19 | <https://apps.who.int/iris/handle/10665/332073> |
| 13-May-20 | Laboratory biosafety guidance related to coronavirus disease (COVID-19), v3 | <https://www.who.int/publications/i/item/laboratory-biosafety-guidance-related-to-coronavirus-disease-(covid-19)> |
| 14-May-20 | Considerations for mass gatherings in the context of COVID-19 (annex to: Considerations in adjusting public health and social measures in the context of COVID-19 | <https://www.who.int/publications/i/item/considerations-for-mass-gatherings-in-the-context-of-covid-19-annex-considerations-in-adjusting-public-health-and-social-measures-in-the-context-of-covid-19> |
| 14-May-20 | Gender and COVID-19 | <https://www.who.int/publications/i/item/gender-and-covid-19> |
| 14-May-20 | Interim guidance for the poliomyelitis (polio) surveillance network in the context of coronavirus disease (COVID-19) | <https://www.who.int/publications/i/item/WHO-POLIO-20.04> |
| 15-May-20 | Multisystem inflammatory syndrome in children and adolescents with COVID-19 | <https://www.who.int/publications/i/item/multisystem-inflammatory-syndrome-in-children-and-adolescents-with-covid-19> |
| 16-May-20 | Cleaning and disinfection of environmental surfaces in the context of COVID-19 | <https://www.who.int/publications/i/item/cleaning-and-disinfection-of-environmental-surfaces-inthe-context-of-covid-19> |
| 18-May-20 | Global COVID-19 Clinical Platform: CRF for suspected cases of multisystem inflammatory syndrome (‎‎‎‎‎MIS)‎‎‎‎‎ in children and adolescents temporally related to COVID-19, v1 | <https://apps.who.int/iris/handle/10665/332121> |
| 18-May-20 | Overview of Public Health and Social Measures in the context of COVID-19 | <https://www.who.int/publications/i/item/overview-of-public-health-and-social-measures-in-the-context-of-covid-19> |
| 19-May-20 | COVID-19 Supply Portal FAQ | <https://www.who.int/publications/m/item/covid-19-supply-portal-frequently-asked-questions> |
| 20-May-20 | Argentina: Prioritizing health for a swift and effective COVID-19 response despite economic fragility and substantial inequalities | <https://www.who.int/publications/m/item/argentina-prioritizing-health-for-a-swift-and-effective-covid-19-response-despite-economic-fragility-and-substantial-inequalities> |
| 20-May-20 | Controlling the spread of COVID-19 at ground crossings | <https://www.who.int/publications/i/item/controlling-the-spread-of-covid-19-at-ground-crossings> |
| 20-May-20 | Somalia: Working with an expanded network of national and international partners to address COVID-19 | <https://www.who.int/publications/m/item/somalia-working-with-an-expanded-network-of-national-and-international-partners-to-address-covid-19> |
| 21-May-20 | Revealing the toll of COVID-19 | <https://www.who.int/publications/i/item/revealing-the-toll-of-covid-19> |
| 22-May-20 | Framework for decision-making: implementation of mass vaccination campaigns in the context of COVID-19 | <https://www.who.int/publications/i/item/WHO-2019-nCoV-Framework_Mass_Vaccination-2020.1> |
| 22-May-20 | Operational planning guidelines to support country preparedness and response, draft as of 22 May 2020 | <https://www.who.int/publications/i/item/draft-operational-planning-guidance-for-un-country-teams> |
| 26-May-20 | Assessment of risk factors for coronavirus disease 2019 (COVID-19) in health workers: protocol for a case-control study (version 1.0) | <https://www.who.int/publications/i/item/assessment-of-risk-factors-for-coronavirus-disease-2019-(covid-19)-in-health-workers-protocol-for-a-case-control-study> |
| 26-May-20 | Population-based age-stratified seroepidemiological investigation protocol for coronavirus 2019 (COVID-19) infection (version 2.0) | <https://apps.who.int/iris/handle/10665/332188> |
| 26-May-20 | Preparing GISRS for the upcoming influenza seasons during the COVID-19 pandemic - practical considerations | <https://apps.who.int/iris/handle/10665/332198> |
| 26-May-20 | Smoking and COVID-19, v1 | https://apps.who.int/iris/handle/10665/332182 |
| 27-May-20 | Clinical management of COVID-19, v5 | [WHO Clinical Management of COVID-19, May 2020](https://www.who.int/publications/i/item/clinical-management-of-covid-19) |
| 28-May-20 | An international randomised trial of candidate vaccines against COVID-19 | <https://www.who.int/publications/i/item/an-international-randomised-trial-of-candidate-vaccines-against-covid-19> |
| 28-May-20 | Ethical considerations to guide the use of digital proximity tracking technologies for COVID-19 contact tracing | <https://www.who.int/publications/i/item/WHO-2019-nCoV-Ethics_Contact_tracing_apps-2020.1> |
| 28-May-20 | Surveillance protocol for SARS-CoV-2 infection among health workers | <https://www.who.int/publications/i/item/WHO-2019-nCoV-HCW_Surveillance_Protocol-2020.1> |
| 29-May-20 | Key planning recommendations for mass gatherings in the context of COVID-19, v3 | <https://www.who.int/publications/i/item/10665-332235> |
| 31-May-20 | Harmonized modules for health facility assessment modules in the context of the COVID-19 pandemic, v1 | <https://apps.who.int/iris/handle/10665/332253> |
| 1-Jun-20 | Global COVID-19 Clinical Platform: CRF for suspected cases of multisystem inflammatory syndrome (MIS) in children and adolescents temporally related to COVID-19, v2 | <https://www.who.int/publications/i/item/WHO-2019-nCoV-MIS_Children_CRF-2020.2> |
| 1-Jun-20 | Operational guidance for the COVID-19 context: Maintaining essential health services, v2 | <https://apps.who.int/iris/handle/10665/332240> |
| 2-Jun-20 | Digital tools for COVID-19 contact tracing (annex to: Contact tracing in the context of COVID-19) | <https://www.who.int/publications/i/item/WHO-2019-nCoV-Contact_Tracing-Tools_Annex-2020.1> |
| 5-Jun-20 | Advice on the use of masks in the context of COVID-19, v4 | <https://apps.who.int/iris/handle/10665/332293> |
| 5-Jun-20 | Monitoring and evaluation framework | <https://www.who.int/publications/i/item/monitoring-and-evaluation-framework> |
| 7-Jun-20 | Medical certification, ICD mortality coding, and reporting mortality associated with COVID-19 | <https://www.who.int/publications/i/item/WHO-2019-nCoV-mortality-reporting-2020-1> |
| 8-Jun-20 | Technical specifications for Pressure Swing Adsorption(PSA) Oxygen Plants | <https://www.who.int/publications/i/item/technical-specifications-for-pressure-swing-adsorption(psa)-oxygen-plants> |
| 11-Jun-20 | Use of chest imaging in COVID-19 | <https://www.who.int/publications/i/item/use-of-chest-imaging-in-covid-19> |
| 11-Jun-20 | Use of chest imaging in COVID-19: web annex A: imaging for COVID-19: a rapid review | <https://apps.who.int/iris/handle/10665/332326> |
| 11-Jun-20 | Use of chest imaging in COVID-19: web annex B: GRADE evidence-to-decision tables | https://apps.who.int/iris/handle/10665/332327 |
| 17-Jun-20 | Criteria for releasing COVID-19 patients from isolation | <https://www.who.int/publications/i/item/criteria-for-releasing-covid-19-patients-from-isolation> |
| 18-Jun-20 | Addressing violence against children, women and older people during the covid-19 pandemic: Key actions | <https://www.who.int/publications/i/item/WHO-2019-nCoV-Violence_actions-2020.1> |
| 20-Jun-20 | Colombia: Through a structured and coordinated response, Colombia seeks to leave no one behind in the fight against COVID-19 | <https://www.who.int/publications/m/item/colombia-through-a-structured-and-coordinated-response-colombia-seeks-to-leave-no-one-behind-in-the-fight-against-covid-19> |
| 23-Jun-20 | Breastfeeding and COVID-19 | <https://www.who.int/publications/i/item/WHO-2019-nCoV-Sci_Brief-Breastfeeding-2020.1> |
| 24-Jun-20 | Critical preparedness, readiness and response actions for COVID-19, v4 | <https://apps.who.int/iris/handle/10665/332665> |
| 25-Jun-20 | Biomedical Equipment for COVID-19 Case Management - inventory tool for facility readiness and equipment re-allocation | https://apps.who.int/iris/handle/10665/332776 |
| 25-Jun-20 | Biomedical equipment for COVID-19 case management - inventory tool: Harmonized health service capacity assessments in the context of the COVID-19 pandemic | <https://www.who.int/publications/i/item/WHO-2019-nCov-biomedical-equipment-inventory-2020.1> |
| 25-Jun-20 | Suite of health service capacity assessments module: Rapid hospital readiness checklist, v1 | <https://apps.who.int/iris/handle/10665/332779> |
| 29-Jun-20 | Infection prevention and control during health care when coronavirus disease (‎COVID-19)‎ is suspected or confirmed, v4 | <https://www.who.int/publications/i/item/WHO-2019-nCoV-IPC-2020.4> |
| 30-Jun-20 | Smoking and COVID-19, v2 | <https://www.who.int/publications/i/item/WHO-2019-nCoV-Sci_Brief-Smoking-2020.2> |
| 6-Jul-20 | Investing in and building longer-term health emergency preparedness during the COVID-19 pandemic | <https://www.who.int/publications/i/item/investing-in-and-building-longer-term-health-emergency-preparedness-during-the-covid-19-pandemic> |
| 9-Jul-20 | Transmission of SARS-CoV-2: implications for infection prevention precautions | <https://www.who.int/publications/i/item/modes-of-transmission-of-virus-causing-covid-19-implications-for-ipc-precaution-recommendations> |
| 10-Jul-20 | Maintaining a safe and adequate blood supply during the coronavirus disease 2019 (COVID-19) pandemic and on the collection of COVID-19 convalescent plasma, v2 | <https://www.who.int/publications/i/item/maintaining-a-safe-and-adequate-blood-supply-during-the-pandemic-outbreak-of-coronavirus-disease-(covid-19)> |
| 10-Jul-20 | WHO Mass gathering COVID-19 risk assessment tool – Sports events | <https://www.who.int/publications/i/item/10665-333187> |
| 11-Jul-20 | WHO Mass gathering COVID-19 risk assessment tool – Religious events | <https://www.who.int/publications/i/item/10665-333186> |
| 12-Jul-20 | WHO Mass gathering COVID-19 risk assessment tool – Generic events | <https://www.who.int/publications/i/item/10665-333185> |
| 13-Jul-20 | Global COVID-19 Clinical Platform: CRF with Pregnancy Module, v5 | <https://www.who.int/publications/i/item/WHO-2019-nCoV-Pregnancy_CRF-2020.5> |
| 13-Jul-20 | Global COVID-19 Clinical Platform: Rapid Case Report Form, v4 | <https://apps.who.int/iris/handle/10665/333229> |
| 17-Jul-20 | Practical actions in cities to strengthen preparedness for the COVID-19 pandemic and beyond | <https://www.who.int/publications/i/item/WHO-2019-nCoV-ActionsforPreparedness-Checklist-2020.1> |
| 20-Jul-20 | Botswana: Bordering Africa’s Epicenter - How early action and careful border control policies have so far contained COVID-19 to clusters | <https://www.who.int/publications/m/item/botswana-bordering-africa-s-epicenter---how-early-action-and-careful-border-control-policies-have-so-far-contained-covid-19-to-clusters> |
| 20-Jul-20 | Kyrgyzstan: Early planning for strong COVID-19 preparedness and response actions | <https://www.who.int/publications/m/item/kyrgyzstan-early-planning-for-strong-covid-19-preparedness-and-response-actions> |
| 23-Jul-20 | Country IAR agenda template | <https://apps.who.int/iris/handle/10665/333772> |
| 23-Jul-20 | Country IAR concept note template | <https://apps.who.int/iris/handle/10665/333774> |
| 23-Jul-20 | Country IAR facilitator's manual | <https://apps.who.int/iris/handle/10665/333773> |
| 23-Jul-20 | Country IAR final report template | <https://apps.who.int/iris/handle/10665/333767> |
| 23-Jul-20 | Country IAR note-taking template | <https://apps.who.int/iris/handle/10665/333768> |
| 23-Jul-20 | Country IAR participant feedback form | <https://apps.who.int/iris/handle/10665/333766> |
| 23-Jul-20 | Country IAR participant feedback form, summary table | <https://apps.who.int/iris/handle/10665/333765> |
| 23-Jul-20 | Country IAR presentation template | <https://apps.who.int/iris/handle/10665/333771> |
| 23-Jul-20 | Country IAR success story template | <https://apps.who.int/iris/handle/10665/333775> |
| 23-Jul-20 | Country IAR trigger question database | <https://apps.who.int/iris/handle/10665/333770> |
| 23-Jul-20 | Guidance for conducting a country COVID-19 intra-action review (IAR) | <https://www.who.int/publications/i/item/WHO-2019-nCoV-Country_IAR-2020.1> |
| 24-Jul-20 | Preventing and managing COVID-19 across long-term care services | <https://www.who.int/publications/i/item/WHO-2019-nCoV-Policy_Brief-Long-term_Care-2020.1> |
| 25-Jul-20 | Safe Eid al Adha practices in the context of COVID-19: Interim guidance | <https://www.who.int/publications/i/item/safe-eid-al-adha-practices-in-the-context-of-covid-19-interim-guidance> |
| 27-Jul-20 | Considerations for implementing mass treatment, active case‐finding and population-based surveys for neglected tropical diseases in the context of the COVID-19 pandemic | <https://www.who.int/publications/i/item/WHO-2019-nCoV-neglected-tropical-diseases-2020-1> |
| 29-Jul-20 | Water, sanitation, hygiene, and waste management for SARS-CoV-2, the virus that causes COVID-19, v4 | <https://www.who.int/publications/i/item/WHO-2019-nCoV-IPC-WASH-2020.4> |
| 31-Jul-20 | GISRS sentinel surveillance for COVID-19 FAQ | <https://apps.who.int/iris/handle/10665/333616> |
| 3-Aug-20 | Considerations for the provision of essential oral health services in the context of COVID-19 | <https://www.who.int/publications/i/item/who-2019-nCoV-oral-health-2020.1> |
| 3-Aug-20 | Reagent calculator for portal | <https://www.who.int/publications/m/item/reagent-calculator-for-portal> |
| 3-Aug-20 | WHO COVID-19 Preparedness and Response Progress Report - 1 February to 30 June 2020 | <https://www.who.int/publications/m/item/who-covid-19-preparedness-and-response-progress-report---1-february-to-30-june-2020> |
| 4-Aug-20 | Estimating mortality from COVID-19 | <https://www.who.int/publications/i/item/WHO-2019-nCoV-Sci-Brief-Mortality-2020.1> |
| 5-Aug-20 | Status of environmental surveillance for SARS-CoV-2 virus | <https://www.who.int/publications/i/item/WHO-2019-nCoV-sci-brief-environmentalSampling-2020-1> |
| 7-Aug-20 | Global surveillance of COVID-19: WHO process for reporting aggregated data, v3 | <https://www.who.int/publications/i/item/WHO-2019-nCoV-surveillance-aggr-CRF-2020.3> |
| 7-Aug-20 | Public health surveillance for COVID-19, v7 | <https://apps.who.int/iris/handle/10665/333752> |
| 7-Aug-20 | WHO COVID-19 Case definitions, v1 | <https://apps.who.int/iris/handle/10665/333912> |
| 12-Aug-20 | Home care for patients with suspected or confirmed COVID-19 and management of their contacts, v4 | <https://www.who.int/publications/i/item/home-care-for-patients-with-suspected-novel-coronavirus-(ncov)-infection-presenting-with-mild-symptoms-and-management-of-contacts> |
| 19-Aug-20 | Considerations for quarantine of contacts of COVID-19 cases, v3 | <https://www.who.int/publications/i/item/considerations-for-quarantine-of-individuals-in-the-context-of-containment-for-coronavirus-disease-(covid-19)> |
| 20-Aug-20 | Malaysia: Strong preparedness and leadership for a successful COVID-19 response | <https://www.who.int/publications/m/item/malaysia-strong-preparedness-and-leadership-for-a-successful-covid-19-response> |
| 21-Aug-20 | Advice on the use of masks for children in the community in the context of COVID-19 (annex to: Advice on the use of masks in the context of COVID-19) | <https://www.who.int/publications/i/item/WHO-2019-nCoV-IPC_Masks-Children-2020.1> |
| 25-Aug-20 | COVID-19 Essential Supplies Forecasting Tool Overview | <https://www.who.int/publications/i/item/WHO-2019-nCoV-Tools-Essential_forecasting-Overview-2020.1> |
| 25-Aug-20 | COVID-19 Essential Supplies Forecasting Tool, v3 | <https://www.who.int/publications/m/item/covid-19-essential-supplies-forecasting-tool> |
| 25-Aug-20 | COVID-19 management in hotels and other entities of the accommodation sector | <https://www.who.int/publications/i/item/operational-considerations-for-covid-19-management-in-the-accommodation-sector-interim-guidance> |
| 25-Aug-20 | Promoting public health measures in response to COVID-19 on cargo ships and fishing vessels | <https://www.who.int/publications/i/item/WHO-2019-nCoV-Non-passenger_ships-2020.1> |
| 27-Aug-20 | Pulse survey on continuity of essential health services during the COVID-19 pandemic | <https://www.who.int/publications/i/item/WHO-2019-nCoV-EHS_continuity-survey-2020.1> |
| 28-Aug-20 | Preventing and managing COVID-19 across long-term care services: Web annex | <https://www.who.int/publications/i/item/WHO-2019-nCoV-Policy_Brief-Long-term_Care-web-annex-2020.1> |
| 2-Sep-20 | Corticosteroids for COVID-19 | <https://www.who.int/publications/i/item/WHO-2019-nCoV-Corticosteroids-2020.1> |
| 3-Sep-20 | The impact of the COVID-19 pandemic on noncommunicable disease resources and services: results of a rapid assessment | <https://www.who.int/publications/i/item/ncds-covid-rapid-assessment> |
| 4-Sep-20 | Infection prevention and control for the safe management of a dead body in the context of COVID-19, v2 | <https://www.who.int/publications/i/item/infection-prevention-and-control-for-the-safe-management-of-a-dead-body-in-the-context-of-covid-19-interim-guidance> |
| 7-Sep-20 | COVID-19 Supply Portal - Supply Coordinators | https://www.who.int/publications/m/item/covid-19-supply-portal-supply-coordinators |
| 9-Sep-20 | WHO Concept for fair access and equitable allocation of COVID-19 health products | <https://www.who.int/publications/m/item/fair-allocation-mechanism-for-covid-19-vaccines-through-the-covax-facility> |
| 11-Sep-20 | Antigen-detection in the diagnosis of SARS-CoV-2 infection using rapid immunoassays | <https://www.who.int/publications/i/item/antigen-detection-in-the-diagnosis-of-sars-cov-2infection-using-rapid-immunoassays> |
| 11-Sep-20 | Diagnostic testing for SARS-CoV-2 | <https://www.who.int/publications/i/item/diagnostic-testing-for-sars-cov-2> |
| 14-Sep-20 | Considerations for school-related public health measures in the context of COVID-19 (annex to: Considerations in adjusting public health and social measures in the context of COVID-19), v2 | <https://www.who.int/publications/i/item/considerations-for-school-related-public-health-measures-in-the-context-of-covid-19> |
| 14-Sep-20 | SAGE values framework for the allocation and prioritization of COVID-19 vaccination | <https://www.who.int/publications/i/item/who-sage-values-framework-for-the-allocation-and-prioritization-of-covid-19-vaccination> |
| 20-Sep-20 | Thailand: How a strong health system fights a pandemic | <https://www.who.int/publications/m/item/thailand-how-a-strong-health-system-fights-a-pandemic> |
| 21-Sep-20 | COVID-19 vaccine introduction readiness assessment tool | <https://www.who.int/publications/i/item/WHO-2019-nCoV-Vaccine-introduction-RA-Tool-2020.1> |
| 21-Sep-20 | Preparing countries for COVID-19 vaccine introduction | <https://www.who.int/publications/i/item/WHO-2019-nCoV-Vaccine-introduction-2020.1> |
| 28-Sep-20 | Target product profiles for priority diagnostics to support response to the COVID-19 pandemic v.1.0 | <https://www.who.int/publications/m/item/covid-19-target-product-profiles-for-priority-diagnostics-to-support-response-to-the-covid-19-pandemic-v.0.1> |
| 30-Sep-20 | Schools and other educational institutions transmission investigation protocol for coronavirus disease 2019 (version 1.1) | <https://www.who.int/publications/i/item/WHO-2019-nCoV-Schools_transmission-2020.1> |
| 1-Oct-20 | Sustainable preparedness for health security and resilience | <https://www.who.int/publications/i/item/9789240015814> |
| 2-Oct-20 | Assessment tool for laboratories implementing SARS-CoV-2 testing, v2 | <https://www.who.int/publications/i/item/assessment-tool-for-laboratories-implementing-covid-19-virus-testing> |
| 2-Oct-20 | Assessment tool for laboratories implementing SARS-CoV-2 testing, v2 | <https://www.who.int/publications/i/item/laboratory-assessment-tool-for-laboratories-implementing-covid-19-virus-testing> |
| 6-Oct-20 | What we know about COVID-19 vaccine development (Coronavirus Update 37) | <https://www.who.int/publications/m/item/what-we-know-aboutcovid-19-vaccine-development> |
| 12-Oct-20 | Handbook for public health capacity-building at ground crossings and cross-border collaboration | <https://www.who.int/publications/i/item/handbook-for-public-health-capacity-building-at-ground-crossings-and-cross-border-collaboration> |
| 13-Oct-20 | Promoting access to Medical Technologies and Innovation, Second Edition, Extract: An integrated health, trade and IP approach to respond to the COVID-19 pandemic | <https://www.who.int/publications/m/item/9789240008267-extract> |
| 15-Oct-20 | Behavioural considerations for acceptance and uptake of COVID-19 vaccines | <https://www.who.int/publications/i/item/9789240016927> |
| 20-Oct-20 | Suite of health service capacity assessments module: Continuity of essential health services: Facility assessment tool, v1 | <https://apps.who.int/iris/handle/10665/336254> |
| 20-Oct-20 | Suite of health service capacity assessments module: Diagnostics, therapeutics, vaccine readiness, and other health products for COVID-19, v1 | <https://apps.who.int/iris/handle/10665/336256> |
| 20-Oct-20 | Suite of health service capacity assessments module: Ensuring a safe environment for patients and staff in COVID-19 health-care facilities | <https://www.who.int/publications/i/item/WHO-2019-nCoV-HCF_assessment-Safe_environment-2020.1> |
| 20-Oct-20 | Suite of health service capacity assessments module: Infection prevention and control health-care facility response for COVID-19 | <https://www.who.int/publications/i/item/WHO-2019-nCoV-HCF_assessment-IPC-2020.1> |
| 22-Oct-20 | Considerations in the investigation of cases and clusters of COVID-19, v3 | <https://www.who.int/publications/i/item/considerations-in-the-investigation-of-cases-and-clusters-of-covid-19> |
| 30-Oct-20 | Prevention, identification and management of health worker infection in the context of COVID-19 | <https://www.who.int/publications/i/item/10665-336265> |
| 1-Nov-20 | Immunization as an essential health service: guiding principles for immunization activities during the COVID-19 pandemic and other times of severe disruption | <https://www.who.int/publications/i/item/immunization-as-an-essential-health-service-guiding-principles-for-immunization-activities-during-the-covid-19-pandemic-and-other-times-of-severe-disruption> |
| 2-Nov-20 | Suite of health service capacity assessments in the context of the COVID-19 pandemic, v2 (update to Harmonized modules for health facility assessment modules in the context of the COVID-19 pandemic from 31 May 2020) | <https://www.who.int/publications/i/item/harmonized-health-service-capacity-assessments-in-the-context-of-the-covid-19-pandemic> |
| 2-Nov-20 | Supporting healthy urban transport and mobility in the context of COVID-19 | <https://www.who.int/publications/i/item/9789240012554> |
| 4-Nov-20 | Considerations for implementing and adjusting public health and social measures in the context of COVID-19, v2 | <https://www.who.int/publications/i/item/considerations-in-adjusting-public-health-and-social-measures-in-the-context-of-covid-19-interim-guidance> |
| 4-Nov-20 | Critical preparedness, readiness and response actions for COVID-19, v5 | <https://www.who.int/publications/i/item/critical-preparedness-readiness-and-response-actions-for-covid-19> |
| 5-Nov-20 | WHO-convened Global Study of the Origins of SARS-CoV-2 | <https://www.who.int/publications/m/item/who-convened-global-study-of-the-origins-of-sars-cov-2> |
| 6-Nov-20 | Readiness for influenza during the COVID-19 pandemic | <https://www.who.int/publications/i/item/WHO-2019-nCoV-Influenza-readiness-COVID-19-2020.1> |
| 8-Nov-20 | Maintaining surveillance of influenza and monitoring SARS-CoV-2 – adapting Global Influenza surveillance and Response System (GISRS) and sentinel systems during the COVID-19 pandemic | <https://www.who.int/publications/i/item/maintaining-surveillance-of-influenza-and-monitoring-sars-cov-2-adapting-global-influenza-surveillance-and-response-system-(gisrs)-and-sentinel-systems-during-the-covid-19-pandemic> |
| 13-Nov-20 | Technical specifications of personal protective equipment for COVID-19 | <https://www.who.int/publications/i/item/WHO-2019-nCoV-PPE_specifications-2020.1> |
| 16-Nov-20 | Guidance on developing a national deployment and vaccination plan for COVID-19 vaccines | <https://www.who.int/publications/i/item/WHO-2019-nCoV-Vaccine_deployment-2020.1> |
| 19-Nov-20 | Temperature-sensitive health products in the Expanded Programme on Immunization cold chain | <https://www.who.int/publications/i/item/WHO-2019-nCov-Immunization-Cold_Chain-2020.1> |
| 20-Nov-20 | Priority medical devices list for the COVID-19 response and associated technical specifications, v2 | <https://www.who.int/publications/i/item/WHO-2019-nCoV-MedDev-TS-O2T.V2> |
| 20-Nov-20 | Suite of health service capacity assessments module: Continuity of essential health services: Facility assessment tool, v2 | <https://www.who.int/publications/i/item/WHO-2019-nCoV-HCF_assessment-EHS-2020.1> |
| 20-Nov-20 | Suite of health service capacity assessments module: Diagnostics, therapeutics, vaccine readiness, and other health products for COVID-19, v2 | <https://www.who.int/publications/i/item/WHO-2019-nCoV-HCF_assessment-Products-2020.1> |
| 20-Nov-20 | Therapeutics and COVID-19, v1 | https://apps.who.int/iris/handle/10665/336729 |
| 25-Nov-20 | Rapid hospital readiness checklist for COVID-19, v2 | <https://apps.who.int/iris/handle/10665/337039> |
| 26-Nov-20 | Suite of health service capacity assessments module: Rapid hospital readiness checklist, v2 | <https://www.who.int/publications/i/item/WHO-2019-nCoV-hospital-readiness-checklist-2020.1> |
| 27-Nov-20 | Evidence to recommendations: COVID-19 mitigation in the aviation sector | <https://www.who.int/publications/i/item/evidence-to-recommendation-covid-19-mitigation-in-the-aviation-sector> |
| 30-Nov-20 | WHO Target Product Profiles for COVID-19 Therapeutics in Hospitalized Patients (October 2020) | <https://www.who.int/publications/m/item/covid-19-therapeutics-target-product-profile-for-hospitalized-patients> |
| 1-Dec-20 | Mask use in the context of COVID-19, v5 | <https://www.who.int/publications/i/item/advice-on-the-use-of-masks-in-the-community-during-home-care-and-in-healthcare-settings-in-the-context-of-the-novel-coronavirus-(2019-ncov)-outbreak> |
| 2-Dec-20 | Generic protocol: A prospective cohort study investigating maternal, pregnancy and neonatal outcomes for women and neonates infected with SARS-CoV-2 (version 2.6) | <https://www.who.int/publications/m/item/a-prospective-cohort-study-investigating-maternal-pregnancy-and-neonatal-outcomes-for-women-and-neonates-infected-with-sars-cov-2> |
| 3-Dec-20 | Health workforce policy and management in the context of the COVID-19 pandemic response | <https://www.who.int/publications/i/item/health-workforce-policy-and-management-in-the-context-of-the-covid-19-pandemic-response> |
| 7-Dec-20 | COVID-19 vaccine introductions and deployment costing tool (CVID tool), version 1.0 first public release (beta) | <https://apps.who.int/iris/handle/10665/337553> |
| 10-Dec-20 | Evidence to recommendations for COVID-19 vaccines: Evidence framework | <https://www.who.int/publications/i/item/WHO-2019-nCoV-SAGE-Framework-Evidence-2020-1> |
| 10-Dec-20 | Public health considerations for elections and related activities in the context of the COVID-19 pandemic | <https://www.who.int/publications/i/item/WHO-2019-nCoV-elections-2020-1> |
| 11-Dec-20 | Checklist to support schools re-opening and preparation for COVID-19 resurgences or similar public health crises | <https://www.who.int/publications/i/item/9789240017467> |
| 16-Dec-20 | Considerations for implementing a risk-based approach to international travel in the context of COVID-19 | <https://www.who.int/publications/i/item/WHO-2019-nCoV-Risk-based-international-travel-2020.1> |
| 16-Dec-20 | COVID-19 diagnostic testing in the context of international travel | <https://www.who.int/publications/i/item/WHO-2019-nCoV-Sci_Brief-international_travel_testing-2020.1> |
| 16-Dec-20 | Public health surveillance for COVID-19, v8 | <https://www.who.int/publications/i/item/who-2019-nCoV-surveillanceguidance-2020.8> |
| 16-Dec-20 | Risk assessment tool to inform mitigation measures for international travel in the context of COVID-19 (annex to: Considerations for implementing a risk-based approach to international travel in the context of COVID-19) | <https://www.who.int/publications/i/item/WHO-2019-nCoV-Risk-based_international_travel-Assessment_tool-2020.1> |
| 16-Dec-20 | WHO COVID-19 Case definitions, v2 | <https://www.who.int/publications/i/item/WHO-2019-nCoV-Surveillance_Case_Definition-2020.2> |
| 17-Dec-20 | Therapeutics and COVID-19, v2 | <https://www.who.int/publications/i/item/therapeutics-and-covid-19-living-guideline> |
| 18-Dec-20 | Apart together survey | <https://www.who.int/publications/i/item/9789240017924> |
| 18-Dec-20 | Emergency Use Designation of COVID-19 candidate vaccines: Ethical considerations for current and future COVID-19 placebo-controlled vaccine trials and trial unblinding | <https://www.who.int/publications/i/item/WHO-2019-nCoV-Policy_Brief-EUD_placebo-controlled_vaccine_trials-2020.1> |
| 18-Dec-20 | SARS-CoV-2 antigen-detecting rapid diagnostic tests: An implementation guide: annex: country readiness checklist for SARS-CoV-2 antigen RDT implementation | <https://apps.who.int/iris/handle/10665/337937> |
| 21-Dec-20 | SARS-CoV-2 antigen-detecting rapid diagnostic tests: An implementation guide | <https://www.who.int/publications/i/item/9789240017740> |
| 22-Dec-20 | Covid-19 vaccines: safety surveillance manual | <https://www.who.int/publications/i/item/10665338400> |
| 22-Dec-20 | SAGE_ Background paper on Covid-19 disease and vaccines (draft prepared by the SAGE Working Group on COVID-19 vaccines) | <https://www.who.int/publications/i/item/background-paper-on-covid-19-disease-and-vaccines> |
| 22-Dec-20 | SAGE_ mRNA vaccines against COVID-19: Pfizer-BioNTech COVID-19 vaccineBNT162b2 (draft prepared by the SAGE Working Group on COVID-19 vaccines) | <https://www.who.int/publications/i/item/mrna-vaccines-against-covid-19-pfizer-biontech-covid-19-vaccinebnt162b2> |
| 23-Dec-20 | Evidence to recommendations: Methods used for assessing health equity and human rights considerations in COVID-19 and aviation | <https://www.who.int/publications/i/item/evidence-to-recommendations-methods-used-for-assessing-health-equity-and-human-rights-considerations-in-covid-19-and-aviation> |
| 23-Dec-20 | Global Risk Communication and Community Engagement Strategy | <https://www.who.int/publications/i/item/covid-19-global-risk-communication-and-community-engagement-strategy> |
| 23-Dec-20 | Rational use of personal protective equipment for coronavirus disease (COVID-19) and considerations during severe shortages, v4 | <https://www.who.int/publications/i/item/rational-use-of-personal-protective-equipment-for-coronavirus-disease-(covid-19)-and-considerations-during-severe-shortages> |
| 23-Dec-20 | Statement of the WHO Working Group on COVID-19 Animal Models (WHO-COM) about the UK and South African SARS-CoV-2 new variants | <https://www.who.int/publications/m/item/statement-of-the-who-working-group-on-covid-19-animal-models-(who-com)-about-the-uk-and-south-african-sars-cov-2-new-variants> |
| 2020 | Checklist on practical actions in cities to strengthen preparedness for the COVID-19 pandemic and beyond | https://apps.who.int/iris/handle/10665/333296 |
| 2020 | Contributions of the polio network to the COVID-19 response: turning the challenge into an opportunity for polio transition | <https://apps.who.int/iris/handle/10665/336261> |
| 2020 | Global spending on health 2020: weathering the storm | <https://apps.who.int/iris/handle/10665/337859> |
| 2020 | Rapid hospital readiness checklist for COVID-19, v1 | <https://apps.who.int/iris/handle/10665/332778> |
| 2020 | Responding to non-communicable diseases during and beyond the COVID-19 pandemic | <https://apps.who.int/iris/handle/10665/334145> |
| 2020 | Responding to non-communicable diseases during and beyond the COVID-19 pandemic: examples of actions being taken by selected members of the United Nations Inter-Agency Task Force on the prevention and control of non-communicable diseases | <https://apps.who.int/iris/handle/10665/334144> |
| 2020 | Responding to non-communicable diseases during and beyond the COVID-19 pandemic: state of the evidence on COVID-19 and non-communicable diseases: a rapid review | <https://apps.who.int/iris/handle/10665/334143> |
| 2020 | Sustaining lives and livelihoods: a decision framework for calibrating social and movement measures during the COVID-19 pandemic | <https://apps.who.int/iris/handle/10665/339598> |
| 2020 | The impact of COVID-19 on mental, neurological and substance use services: results of a rapid assessment | <https://apps.who.int/iris/handle/10665/335838> |
