# Supplementary material for: A gendered content analysis of the World Health Organization’s COVID-19 guidance and policies
Source: PLOS Glob Public Health. 2022 Jun 30;2(6):e0000640. doi: 10.1371/journal.pgph.0000640 (PMC10021261; doi:10.1371/journal.pgph.0000640)
Supplement: Attachment — Submitted filename: Response to Reviewers.docx [file pgph.0000640.s002.docx]

Dear Editors and Reviewers,

Thank you for taking the time to review and consider our paper entitled "A gender analysis of the World Health Organization’s COVID-19 guidance and policies”, for publication with Plos Global Public Health. We really appreciate your ongoing support and feedback, particularly amid the onslaught of pressures that people are facing on their time during the pandemic.

We have responded to your points below in bold.

**Editor’s comments**

This is an important policy analysis on considerations of sex and gender in WHO policy documents. One of the issues that I observed while reviewing this paper was how to best understand the ways in which actors should utilize terms such as “women and girls”. My understanding from reading this piece is that solely focusing on “women and girls” deemphasizes the focus on sexual minorities and gender minorities (p. 9, where the authors call the primary use of this term problematic); however, the authors use women and girls periodically throughout the manuscript (for example, p. 23 or women and gender noted on p. 21) to discuss the lack of gender-mainstreaming in WHO’s policy documents. It would be important to clarify the authors’ stance so that readers are clear on where the gaps lie.

**We have both changed the phrasing on pages 10, 21, and 23 to be more inclusive, using language from the definition of gender-mainstreaming, and eliminating our use of the term “women and girls” outside discussing WHO using it. We have also included in the limitation section (new) the challenges of the women and girls focus with understanding gender in global health.**

Please provide additional details on the content analysis – please see Reviewer 1’s suggestions on this.

**See below.**

In the Results section, there appeared to be inconsistent use of citations for documents – please review carefully to make sure that documents are cited wherever feasible. Some of the quotes can be reduced in length.

**We have included more citations and shortened some of the quotes to ensure greater consistency**

R1 makes an important point about providing some additional depth in the analysis in terms of what these findings mean for issues of power in policy and decision-making processes within global health organizations; I recognize that this is not the primary objective of the paper, but the authors could provide some limited additional analysis in this direction in order to strengthen the discussion.

**See below**

**Reviewer’s comments**

Thank you for the opportunity to review this paper, which contributes to the literature in term of how CVDID-19 has made more ‘visible’, pre-existing gender inequalities and the need for detailed gender analyses of policy and programmes.

Comments and questions below are intended to strengthen the paper:

**Introduction:**

- In terms of the quote below, how and why this has this definition come to be, As would be a very insightful finding g finding in and of itself?

**Thank you for this important question – however, we do not know how this definition of WHO’s gender mainstreaming has come to be, and indeed, a literature and policy search to try and answer this question has surmounted to nothing. This is the WHO’s position, with their limited gender team, and so we take it at face value. Whilst we agree there would be an interesting piece of research to trace the development of gender understanding and policy within WHO, this is beyond the scope of this paper, which is focused on how WHO implemented their commitment to gender mainstreaming (as listed here) in the context of COVID19 policies.**

- Can you say more about technical versus political processes as part of the analysis should include what the policy process factors are that contributed to such a narrow definition and framing

**We have added to the discussion in order to link back to this question in an attempt to explain, within the scope of the paper, the narrow definition, framing, and therefore implementation of gender mainstreaming.**

- Was intersectionality considered at all?

**By the WHO? The short answer is no.**

**In our analysis? The short answer is ‘not really’.**

**We recognise that it is a limitation of our paper, and indeed of WHO’s work in this area, that it doesn’t consider intersectional factors in how gender is experienced and indeed how policies are created and integrated. We performed a gendered policy analysis, rather than intersectional analysis – we have now updated the paper to include this as a limitation in the discussion.**

“The WHO’s definition of gender mainstreaming (above) reflects a focus on process (i.e. “assessing implications” as opposed to rectifying inequities) and on “technical areas and levels”, as opposed to political processes or power relationships. Recent analysis has further revealed tensions between the formal commitment to gender mainstreaming within WHO, and the failure to implement practices of gender mainstreaming throughout programmes [4]. Our analysis therefore takes this narrow definition of gender mainstreaming as a baseline, asking first if the WHO met its own standards of gender mainstreaming within theCOVID-19 response, while keeping in mind calls for more transformative approaches.

**Methods**

- Please provide details on who did the analysis of the policy documents, was it conducted by one person, 2 or 3 independent reviewers?

**We have added a paragraph to this effect, to ensure that we were clear in the verification and review processes we undertook – one researcher conducted the bulk of the downloading and analysis, but a random selection were moderated by the rest of the team to ensure that there was consistency and agreement in the coding and analysis being undertaken**

- How was agreement reached between the reviewers who conducted the analysis? What happened when the reviewers could not agree, how was consensus reached?

**The team had continual discussions throughout the process to iteratively determine whether there were any borderline or gray areas, and whether or how to include them. There were no disagreements after these discussions. All authors approved the final analysis framework.**

- Did you exclude and policy documents, and was the exclusion criteria?

**We had already included the inclusion and exclusion criteria in the paper: We searched all “COVID_19” tagged publications on the website and in the WHO Publications Repository. Publications were limited to one year (2020) and included policy and advocacy briefs, health advisories, technical guidance, interim guidance, scientific briefs, country missions, survey reports, research frameworks, case report forms (CRFs), and various surveillance tools and user guides. Joint publications co-authored by the WHO and other organizations were included. WHO regional office publications, country-specific situation reports and status updates, weekly epidemiolocal updates, journal articles, and corrigenda were excluded.**

- What theory and informed your content analysis?

Pope, C., Ziebland, S., Mays, N., Bmj, S., Medical, B., Jan, N., … Pope, C. (2017). Analysing qualitative data, *320*(7227), 114–116.

Dalglish, S. L., Khalid, H., & McMahon, S. A. (2020). Document analysis in health policy research: the READ approach. *Health Policy and Planning*, 1–8. https://doi.org/10.1093/heapol/czaa064

**Thank you for nudging us to be more explicit on this – we used the READ approach you have highlighted above and have ensured that this is now clear throughout the methods section, and indeed referenced.**

- An analysis of policy is not just content of policy documents, but about the actors, context and processes that shaped those in a dynamic way. What can be said about the role of actors, context, and processes from a gender perspective?
- Even though the focus was on policy documents these broader elements need to be described and acknowledged in both the methods and the findings

See for example the work of Gilson and others from filed of Health Policy Analysis

Gilson, L., Orgill, M., & Shroff, Z. C. (2018). *A Health Policy Analysis Reader: the Politics of Policy Change in Low-and Middle-Income Countries*. Retrieved from https://www.who.int/alliance-hpsr/resources/publications/Alliance-HPA-Reader-web.pdf

Jacobs, T., George, A. S., & De Jong, M. (2021). Policy foundations for transformation – a gender analysis of adolescent health policy documents in South Africa. *Health Policy and Planning*, 1–11. <https://doi.org/10.1093/heapol/czab041>

Mukuru, M., Kiwanuka, S. N., Gilson, L., Shung-King, M., & Ssengooba, F. (2020). “The Actor Is Policy”: Application of Elite Theory to Explore Actors’ Interests and Power Underlying Maternal Health Policies in Uganda, 2000-2015. International Journal of Health Policy and Management, (x), 1–14. <https://doi.org/10.34172/ijhpm.2020.230>

Baker, P., Friel, S., Kay, A., Baum, F., Strazdins, L., & Mackean, T. (2018). What enables and constrains the inclusion of the social determinants of health inequities in government policy agendas? A narrative review. *International Journal of Health Policy and Management*, *7*(2), 101–111. <https://doi.org/10.15171/ijhpm.2017.130>

McDougall, L. (2016). Power and Politics in the Global Health Landscape: Beliefs, Competition and Negotiation Among Global Advocacy Coalitions in the Policy-Making Process. *International Journal of Health Policy and Management*, *5*(5), 309–320. <https://doi.org/10.15171/ijhpm.2016.03>

**We understand that policy is not made in a vacuum, but that decisions and pathways are made by actors which have an identity and agenda, as well as in a particular place and time, and by particular leaders. Each of these are vital to understand, and we have sought to unpack these in our discussion, as encouraged by the editor. We haven’t included heavy incursion of this as this is not the main aim of the paper, and nor do we have the data to trace the policy development to understand the process, for example, we didn’t conduct interviews with the policymakers to understand how these came about. However, we have expanded the discussion to ensure that the WHO as an actor and contemporary debates about gender in global health, as well as the role of the WHO as a norm entrepreneur are included to try and bring this important point more fully to the fore (that we cannot separate policies from those who make them).**

**Results**

- The results describe what there is explicitly across the themes, but the analysis can be deepened by thinking about what is said more implicitly i.e. how does this reflect the ideas of actors, policy process and what the implications are of such a framings. Please add more detail

**As per the above and comments from the editor, we have sought to do so in the discussion rather than in the results, as our methodology does not enable us to have such content in the results, only as part of the broader “distilling” of the results amid the context.**

- A gender analysis importantly needs to focus on the recognition and analysis of power, particularly how power is wielded and distributed given that gender relations, are all about power. Please add this in as a way of strengthening the paper.

**We agree completely and one of the findings of our analysis, which we have clarified, is the lack of recognition of the power dynamics related to health inequities by the WHO, and the need for greater consideration of these.**

In your discussion you mention “The WHO might seize the opportunity created

by COVID-19, and its starkly gendered effects, to not only reaffirm its commitment to gender mainstreaming, but shift to a more transformative approach that includes acting on, as well as assessing, gender inequities,”

This describes the *what*, but more of a deeper analysis could explain the *why*

and could address some of the following questions:

**We have added much into the discussion to further the “why” – notably a paragraph at the start to insinuate why we think WHO may not be thinking about gender in its covid-19 policymaking. However, this is not based on data, but on our knowledge and literature of the organization, and thus it remains speculative, albeit referenced.**

- What are the implications of the results for structural shifts in global health systems?

**We do not consider this (or indeed ourselves) to be a health systems research(ers) per se, we believe this to be beyond the scope to incorporate.**

- How can the results be used as an opportunity to further foreground gender inequality as critical element of health sector reform?

**This is an important point, and our data can help to highlight the lack of gender across the organisation’s policies, and the need to counteract this as part of WHO reform efforts which are ongoing – as a paper is on WHO we prefer to consider this reform, rather than broader health sector reform. We have included this in the discussion accordingly.**

- Given global context of regressive changes what are the potential backlashes and how could these be managed?

**We have made reference to the right wing tendencies of many governments during COVID, particularly when this comes to gender, but as we do not have data on member states and/or their impact on covid policies at WHO we do not feel we can elaborate on this point too much.**

- What are your reflections on WHO as a patriarchal and gendered institution?

**The WHO, like all organisations have formal and informal pathologies for the internal governance of the institution, and this can be seen in how gender policies are implemented. Formally they may have committed to gender mainstreaming across their work, but our paper demonstrates that this has not been wholesale across the policies and that this commitment has not trickled down, either informally or formally. It would be interesting to know why gender did come in some instances – is this because some policymakers as individuals are more informally engaged – but we do not have the data to validate such a claim. There may be multiple reasons, but one such reason may be the informal pathology of the institution which is gender blind, or too focused on the biomedical to see the downstream social effects of policies. This can be true both of gender, or indeed any equity concern. As has been argued elsewhere (e.g. Wenham&Davies 2021), WHO has a gender problem, and there remains significant gaps in their implementation of a meaningful gender policy. Our paper helps to quantify and qualify such an assertion, confirming the gender blindness within the institution.**

- In your view, what are the implications of these results on health systems building blocks?

**Thank you for asking about building blocks – as we do not consider this (or indeed ourselves) to be a health systems research per se, we believe this to be beyond the scope to incorporate.**

- Transforming gender inequality is complex and multi-dimensional and what other considerations beyond policy change are needed to lay the foundations for change?

**This is clearly a big answer – we need policy change, leadership engagement, champions across all levels of the institution, institutional structural changes to facilitate greater gender engagement, push and agreement from member states to accept such changes, greater civil society involvement, awareness of intersectoral issues etc – this could be a paper in and of itself, and nor do our data help to answer such a question, as such we have not included this in our revision, as it would be speculative.**

- What are the organisational processes and coordination within WHO department that is needed e.g., Gender, Equity and Rights and those departments focussed on SRHR as well as with the UN agencies? What are the leadership and management implications at WHO? Describe the actors and who needs to lead change within and beyond WHO?

**This is an important point, but our methodology leads itself to analyse the outputs (policy) rather than the processes. As we only looked at the policies in 2020 during the height of the pandemic initially, we are unable to understand policy tracing exercises to see how the policy may have development akin to different leadership or contemporary discourse. Moreover, as we didn’t undertake interviews with policymakers it is hard to understand intentions or limitations. We have added this to a limitation sections as we recognise the gap this has for the research and findings.**
