# Supplementary material for: A gendered content analysis of the World Health Organization’s COVID-19 guidance and policies
Source: PLOS Glob Public Health. 2022 Jun 30;2(6):e0000640. doi: 10.1371/journal.pgph.0000640 (PMC10021261; doi:10.1371/journal.pgph.0000640)
Supplement: Attachment — Submitted filename: PLOS_PGPH_Response2Review2.docx [file pgph.0000640.s003.docx]

Dear Dr Sriram

Thank you for the opportunity to further revise our manuscript and your helpful comments. We have responded to your and the reviewer’s comments in detail below. Please let us know if you need any further information.

Sincerely,

Julia

*Journal requirements*

1. Please change "Figure one" to "Fig 1" where you refer your figure on page 9.

- this is changed

2. Please review your reference list to ensure that it is complete and correct. If you have cited papers that have been retracted, please include the rationale for doing so in the manuscript text, or remove these references and replace them with relevant current references. Any changes to the reference list should be mentioned in the rebuttal letter that accompanies your revised manuscript. If you need to cite a retracted article, indicate the article’s retracted status in the References list and also include a citation and full reference for the retraction notice.

- we have doubled checked the reference list

*Additional Editor Comments (if provided):*

- R1 notes that the authors should have a deeper discussion of social and political processes - my sense is that the authors have addressed this concern within the boundaries of the paper. I would suggest reviewing the reviewer’s feedback on this point and suggested papers to see if there are any other places within the manuscript to strengthen this particular concern.

- Thank you for recognizing we have aimed to address this concern within the boundaries of the paper. We have made some slight changes in response to the reviewer’s comments.

- Page 6 – should be Microsoft Excel instead of excel?

- yes, we have made this edit

- Page 23 – The point about right wing populism in 2020 – my sense is that there is a longer trajectory of that trend that predates 2020, and so would suggest reframing.

- We have decided to delete this sentence as is unnecessary

-Please consider R1’s suggestion about citing Ravindran et al 2021.

- we have cited Ravindran et al. 2021

*Reviewer’s comments*

Thank you for the opportunity to further review this paper, and your detailed consideration has strengthened the paper. I think you have integrated most of them well, however 2 have been insufficiently incorporated:

1.

- An analysis of policy is not just content of policy documents, but about the actors, context and processes that shaped those in a dynamic way. What can be said about the role of actors, context, and processes from a gender perspective?
- Even though the focus was on policy documents these broader elements need to be described and acknowledged in both the methods and the findings.

We agree that policy analysis as a whole includes consideration of actors, context and processes. However, the paper’s objective is to conduct a content analysis of policy documents, and we are cautious about deviating from our focus, particularly considering the need to keep to a word limit etc. Moreover, we do not feel that our methodology, based on content documentary analysis allows us to explore the actors, context and processes in the same way. For that we believe we would need to add in additional interviews, process tracing, organizational ethnographies and/or participant observation. As such, we feel limited to what extent we can elaborate on these dynamic factors. That said we have aimed to emphasis the role of the actor (WHO) where we are able to justify the intervention and the particularities of the context (COVID-19 pandemic) and related processes within the institution we have the data for, while clarifying the limits of the paper for not being able to do this to the full extent. See for example the slight changes in the methods and discussion sections, as well as the already existing discussion on the context of the WHO in global health governance, the waning power of the actor during the COVID-19 pandemic and its historic gender relations.

- see you have incorporated the work of Carol Bacchi which is great, but further recognition of the social and political processes that construct policy documents are essential, even if you only focussed on the documents themselves. See also Bacchi, C. (2016). Problematizations in Health Policy: Questioning How “Problems” Are Constituted in Policies. SAGE Open, 6(2). <https://doi.org/10.1177/2158244016653986>
- It is challenging to integrated Bacchi’s and similar post structural approaches as our content analysis takes a very different, more critical realist approach. However, we recognize the value of Bacchi’s work and have referenced the paper above in the discussion around how risk communications is represented as a problem.

And as mentioned before, see for example the work of Gilson and others from field of Health Policy Analysis

Gilson, L., Orgill, M., & Shroff, Z. C. (2018). *A Health Policy Analysis Reader: the Politics of Policy Change in Low-and Middle-Income Countries*. Retrieved from https://www.who.int/alliance-hpsr/resources/publications/Alliance-HPA-Reader-web.pdf

Jacobs, T., George, A. S., & De Jong, M. (2021). Policy foundations for transformation – a gender analysis of adolescent health policy documents in South Africa. *Health Policy and Planning*, 1–11. <https://doi.org/10.1093/heapol/czab041>

Mukuru, M., Kiwanuka, S. N., Gilson, L., Shung-King, M., & Ssengooba, F. (2020). “The Actor Is Policy”: Application of Elite Theory to Explore Actors’ Interests and Power Underlying Maternal Health Policies in Uganda, 2000-2015. International Journal of Health Policy and Management, (x), 1–14. <https://doi.org/10.34172/ijhpm.2020.230>

Baker, P., Friel, S., Kay, A., Baum, F., Strazdins, L., & Mackean, T. (2018). What enables and constrains the inclusion of the social determinants of health inequities in government policy agendas? A narrative review. *International Journal of Health Policy and Management*, *7*(2), 101–111. <https://doi.org/10.15171/ijhpm.2017.130>

McDougall, L. (2016). Power and Politics in the Global Health Landscape: Beliefs, Competition and Negotiation Among Global Advocacy Coalitions in the Policy-Making Process. *International Journal of Health Policy and Management*, *5*(5), 309–320. https://doi.org/10.15171/ijhpm.2016.03

- Thank you for this additional list – we have incorporated Jacobs et al and McDougall into the paper.

2.

- Transforming gender inequality is complex and multi-dimensional and what other considerations beyond policy change are needed to lay the foundations for change? How would recommend gender be mainstreamed in WHO. COVID has made visible how gender inequalities are exacerbated by other inequalities. How should these be considered by WHO?
- We have added to our recommendations in the discussion to further answer these questions, or point readers to works that look beyond policy change.

See for examples the work Ravindran et al cited below:

Ravindran, T. K. S., Ippolito, A. R., Atiim, G., & Remme, M. (2021). Institutional gender mainstreaming in health in UN Agencies: Promising strategies and ongoing challenges. Global Public Health.

https://doi.org/10.1080/17441692.2021.1941183

- Thank you for this recommendation, we have cited this article.
